# Atrial fibrillation post hematopoietic stem cell transplant: incidence, risk factors, and management challenges

**DOI:** 10.3389/fcvm.2026.1781484

**Published:** 2026-05-13

**Authors:** Hassan Kawtharany, Mohamad Ghazal, Abdallah Rayyan, Faizi Jamal

**Affiliations:** 1 Department of Internal Medicine, Saint Louis University School of Medicine, St Louis, MO, United States; 2 Internal Medicine Department, Albany Medical Center, Albany, NY, United States; 3 College of Medicine, University of Central Florida/HCA North Florida Internal Medicine Department, Gainesville, FL, United States; 4 Division of Cardiology, Department of Medicine, City of Hope National Medical Center, Duarte, CA, United States

**Keywords:** allogenic hematopoietic cell transplantation (alloHCT), atrial fibillation, autologous hematopoeitic cell transplant, bone marrow transplant (BMT), hematopoetic stem cell transplant, stroke

## Abstract

Atrial fibrillation (AF) is the most frequent cardiovascular complication after hematopoietic stem cell transplantation (HSCT) and is linked to higher short-term and long-term morbidity and mortality. AF occurring within 30 days post-HSCT reflects an interplay of peri-transplant triggers, conditioning-related myocardial injury, systemic inflammation and infection, rapid fluid shifts with atrial stretch, and electrolyte disturbances. Beyond 30 days, AF is driven by chronic inflammation and graft-vs.-host disease, metabolic dysregulation from immunosuppression, and cumulative cardiotoxic cancer therapies. Management is challenged by thrombocytopenia and drug–drug interactions. In the acute phase, meticulous volume and electrolyte optimization is essential. When AF duration is ≤48 h, preferably ≤12 h, rhythm control (often with amiodarone) may be pursued; otherwise, rate control is generally favored, with beta-blockers preferred. Long-term care should individualize rate vs. rhythm strategies and reassess anticoagulation as counts recover, given potential thromboembolic risk. Prospective studies and HSCT-specific AF risk tools are needed to guide prevention and treatment.

## 1 Background and incidence

Hematopoietic stem cell transplantation (HSCT) is a potentially curative treatment for a broad range of malignant and non-malignant hematologic disorders (1, 2). By enabling the delivery of high-dose chemotherapy and/or radiotherapy followed by hematopoietic rescue, HSCT, whether performed as autologous or allogeneic, has transformed outcomes for patients with otherwise poor prognoses (1). Advances in conditioning regimens, donor selection, supportive care, and infection prophylaxis have markedly improved survival, resulting in a growing population of long-term HSCT survivors (3). In the United States, the number of survivors is projected to rise from approximately 242,000 in 2020 to over 500,000 by 2030 (4, 5).

With increased survivorship has come growing recognition of cardiovascular complications as a major cause of morbidity and mortality in HSCT recipients, paralleling trends observed across the broader cancer survivorship population. Cardiovascular disease in this setting arises from a complex interplay of preexisting patient related risk factors, cumulative cardiotoxic exposures (e.g., anthracyclines, high-dose cyclophosphamide, chest radiation), transplant-related inflammation, metabolic derangements, immune-mediated vascular injury, and accelerated cardiovascular aging, particularly in individuals with limited cardiovascular reserve (5–7). Cardiovascular complications after HSCT are often classified as short-term and long-term using the 100-day cutoff (8, 9). Alternatively, some studies propose categorizing them based on timing relative to transplant: the transplant phase (within 30 days), the immediate post-transplant phase (30–100 days), and the late post-transplant phase (>100 days), which encompasses survivorship complications (10).

Among these complications, atrial fibrillation (AF), the most common sustained cardiac arrhythmia in the general population (11), has emerged as the most frequent cardiovascular event after HSCT (5, 9, 12). In a meta-analysis of 13 studies (*n* = 10,587), AF was the most common arrythmia post HSCT with a pooled estimated incidence of AF within 30 days following HSCT of 4.2% (95% CI: 1.7%–9.6%) (12). AF reported incidence varies depending on follow-up duration, underlying population, transplant type, and methods of detection, but consistent temporal patterns are observed across studies. In the early post-transplant period (≤100 days), AF incidence generally ranges from approximately 2% to 6% in large contemporary cohorts. Higher early incidence has been reported in a contemporary multiple myeloma cohort, reaching approximately 5.5%, likely reflecting the older age of this population and longer median follow-up duration (13). Beyond the acute phase, AF incidence increases progressively over time. Across longitudinal cohorts, cumulative incidence reaches approximately 4%–7% at 1 year, 7%–10% at 5 years, and ∼10% at 10 years (7, 9, 13). Importantly, AF incidence appears broadly comparable between autologous and allogeneic HSCT across both short- and long-term follow-up. Higher incidence rates in Chang et al. and Bolaji et al. compared with Vasbinder et al., are likely attributable to variations in follow-up duration, with longer median follow-up enabling more complete event capture and less loss to follow-up (7, 9, 13). A summary of studies reporting AF incidence following HSCT is provided in Table 1 (7, 9, 13–22).

**Table 1:** Characteristics of studies reporting on incidence of AF post HSCT.

Author	Country, year	Study design	Population	Gender (% male)	Age (years)	Transplant Type	Atrial fibrillation/Total (%)	Follow up duration
Hidalgo et al	USA, 2004	Retrospective single-center analysis	Lymphoma (54%), Leukemia (18%), Breast Cancer (14%), MM (12%), MDS (2%)	60%	Median (range): 59 (19–70)	Autologous (75%), Allogeneic (25%)	45/ 1,465 (3%)	Median 188 days
Fatema et al	USA, 2009	Retrospective review of registry	MM	56%	Mean (SD): 57 (9)	Autologous	39/ 383 (10.2%) (39 per 1,000 person-years)	Median 2.6 years (total 1,002 person-years)
Peres et al	USA, 2010	Retrospective chart analysis	NHL (8–17%), CLL (8–12%), AML/MDS (20–24%)	68%	Median (range): 56 (42–68)	Allogenic	17/278 (6%)	100 days
Motoki et al	Japan, 2010	Retrospective study	Leukemia (ALL and AML)	50%	Mean (SD) 6.9 ± 3.5 years	Allogeneic	0/18 (0%)	∼2 months (to discharge)
Sureddi et al	USA, 2012	Retrospective review	MM	72%	Mean (SD): 64.4 ± 8.1 years	Autologous	75/278 (27%)	NR
Steuter et al	USA, 2013	Retrospective case-control study	NHL (63%–77%), HD (1%–2%), MM (20%–36%)	66%	Median (range): 63 (50–72)	Autologous	44/516 (8.5%)*AF/AFT	100 days post-transplant
Singla et al	USA, 2013	Single-center retrospective cohort	Plasma cell disorders (MM, Amyloidosis, POEMS), Lymphoma, Leukemia	61%	Median (range) 58 (19–77)	Autologous	71/983 (7%)	Median 19–22 days
Tonorezos et al	USA, 2015	Retrospective single-center analysis	Lymphoma (36%), MM (28%), Leukemia (20%), MDS (6%)	60%	Median (range): 56 (40–76)	Autologous (64%), Allogeneic (36%)	30/1,177 (2.5%)	1 year
Chang et al	USA, 2021	Retrospective single center cohort	AML, ALL, MDS, MPN	57.7%	Median (range): 52.4 (18.1–78.6)	Allogeneic	1 year: 33/487 (6.8%) 5 years: 52/487 (10.6%)	Median 3.1 years
Vasbinder et al-autologous	USA, 2023	Multicenter retrospective cohort	MM, DLBCL, HL	59.1%	Mean (SD): 56.6 (12.8)	Autologous	100-day: 56/2004 (2.8%) 1 year: 72/2004 (3.6%) 5 years: 134/2004 (6.7%) 10 years: 211/2004 (10.55%)	Median 2.3 years
Vasbinder et al-allogenic	USA, 2023	Multicenter retrospective cohort	AML, ALL, MDS	59.1%	Mean (SD): 52.3 (13.8)	Allogeneic	100-day: 31/1,350 (2.3%) 1 year: 49/1,350 (3.6%) 5 years: 93/1,350 (6.9%) 10 years: 128/1,350 (9.5%)	Median 2.3 years
Aghel et al	Canada, 2025	Single-center retrospective cohort study	AML, ALL, MDS, Myelofibrosis, Lymphoma, CLL, CML, CMML	58.57%	Median (range): 54 (17–76)	Allogeneic	33/852 (3.92%)*AF/AFL	100 days
Bolaji et al	USA, 2026	Retrospective cohort analysis	MM	58.7%	Median (range): 62 (24–81)	Autologous	90 days: 44/801 (5.5%) 3 years: 72/801 (9%)	Median 36.2 months

AF, atrial fibrillation; AFL, atrial flutter; HSCT, hematopoietic stem cell transplantation; MM, multiple myeloma; NHL, non-hodgkin lymphoma; HD, hodgkin disease; AML, acute myeloid leukemia; ALL, acute lymphoblastic leukemia; MDS, myelodysplastic syndrome; MPN, myeloproliferative neoplasm; DLBCL, diffuse large B-cell lymphoma; CLL, chronic lymphocytic leukemia; CML, chronic myeloid leukemia; CMML, chronic myelomonocytic leukemia; NR, not reported; SD, standard deviation.

The occurrence of AF following HSCT has been consistently associated with worse outcomes, including increased short-term and long-term mortality, non-relapse mortality (NRM), and acute cardiovascular complications (5, 7, 13, 22). Despite heterogeneity in patient populations and follow-up durations (ranging from 3 months to 5 years), the magnitude of risk is consistently large, with studies reporting approximately 3- to 15-fold increases in all-cause mortality and NRM. In an acute leukemia predominant cohort, AF was associated with a 12.8-fold higher risk of all-cause mortality and a 15.8-fold higher risk of NRM in allogeneic HSCT recipients over 5 years of follow-up (7). Similarly, in predominantly acute leukemia population, Aghel et al. reported an approximately sixfold increase in both all-cause mortality and NRM within 100 days (22). Consistent findings have also been observed in a multiple myeloma cohort, where AF was associated with a fivefold increase in all-cause mortality and a 4.5-fold increase in NRM over a median follow-up of 3 years (13). Collectively, these findings suggest that AF in the HSCT setting is not merely an isolated arrhythmic event but may serve as a prognostic marker of adverse outcomes, reflecting systemic vulnerability and impaired cardiovascular reserve. An important unresolved question, however, is whether AF directly contributes to NRM or instead reflects underlying frailty and physiologic stress (23).

Given its high prevalence, prognostic significance, and potential for complicating already complex clinical management, including rate/rhythm control and anticoagulation decisions in thrombocytopenic or medically fragile patients, AF represents a critical and under-recognized challenge in the cardio-oncology care of patients with HSCT. The purpose of this review is to synthesize current evidence on the epidemiology, risk factors, and clinical outcomes of AF in HSCT recipients; explore underlying mechanisms; discuss management strategies; and highlight research gaps.

## 2 Mechanisms of atrial fibrillation in the setting of hematopoietic stem cell transplantation

**Figure 1:**
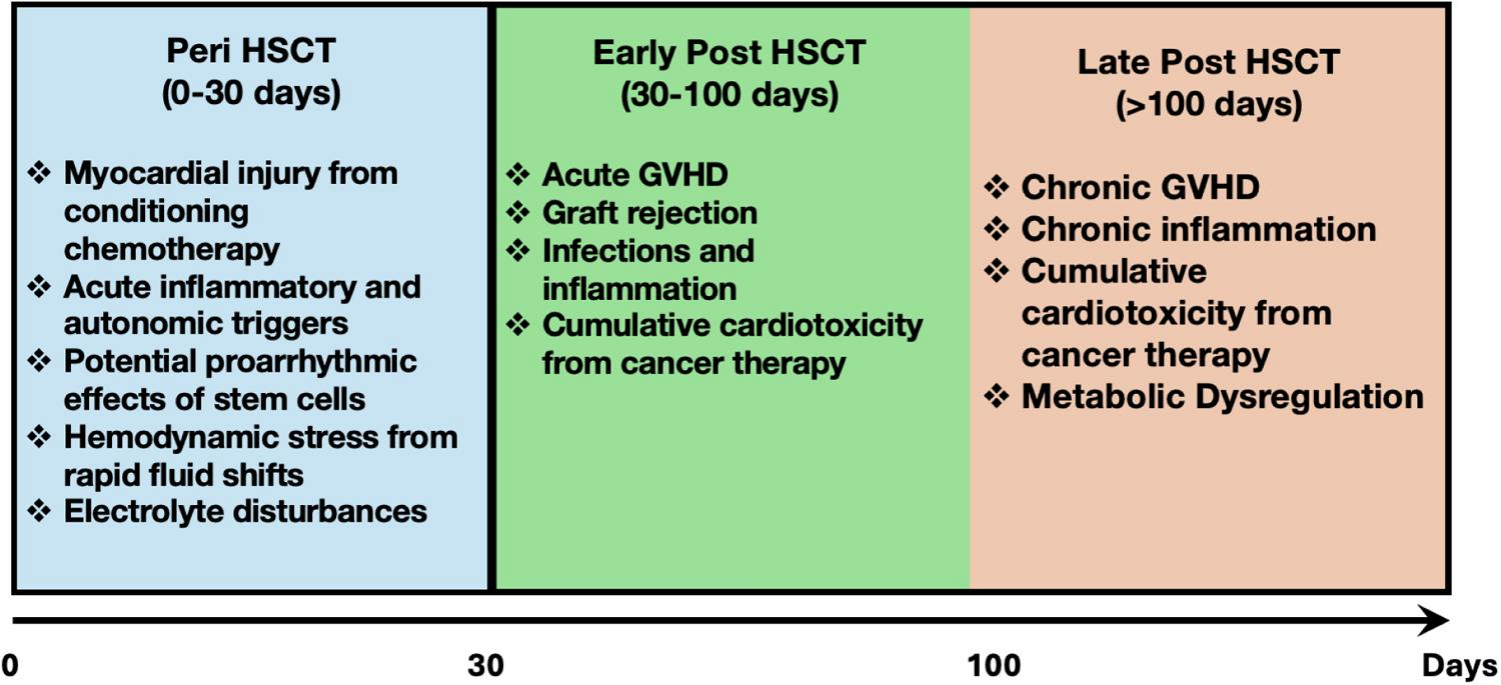
Mechanisms of new onset atrial fibrillation in the peri- and post-HSCT period. HSCT, hematopoietic stem cell transplant; GVHD, graft versus host disease.

AF following HSCT reflects an interplay of pre-existing cardiovascular risk factors, direct cardiotoxic effects of conditioning regimens, acute peri-transplant physiological stressors and transplant-related complications. The pathophysiology is multifactorial, with contributions from structural, electrophysiological, and neurohormonal changes that create a proarrhythmic substrate. Understanding these mechanisms requires considering both peri-HSCT factors, occurring during the immediate conditioning and engraftment period (within 30 days) and post-HSCT factors, which emerge after 30 days (immediate post-HSCT) and during long-term recovery and survivorship (after 100 days) (Figure 1).

### 2.1 Peri- HSCT factors (within 30 days)

#### 2.1.1 Myocardial injury from conditioning chemotherapy

Conditioning regimens for HSCT differ by transplant type but often include high-dose alkylating agents such as melphalan, cyclophosphamide, or busulfan, each capable of inducing myocardial injury and predisposing to AF. Melphalan, frequently used in autologous HSCT for multiple myeloma, has been associated with supraventricular arrhythmias in up to 11% of patients (predominantly AF), a rate significantly higher than with other regimens (24). Its cardiotoxicity is linked to increased oxidative stress, which disrupts calcium handling, impairs contractility, and alters expression of key contractile proteins, collectively promoting arrhythmogenesis (25). Cyclophosphamide, commonly incorporated into conditioning regimens, can cause cardiotoxicity through direct endothelial and myocardial injury mediated by toxic metabolites and oxidative stress, resulting in myocyte apoptosis and interstitial hemorrhage that predispose to tachyarrhythmias such as atrial fibrillation (26, 27). Busulfan, often combined with cyclophosphamide for leukemia conditioning, has been reported to cause AF in up to 6.4% of cases (27), while its exact mechanism remains unclear, endothelial and sinusoidal injury with resultant fluid retention and atrial stretch are suspected contributors.

#### 2.1.2 Acute inflammatory and autonomic triggers

The peri-transplant period is characterized by profound immunosuppression and neutropenia, increasing the risk of infection. Febrile neutropenia, and sepsis can increase sympathetic tone and catecholamine release, creating a potent trigger for AF (28). Additional stressors, such as pain, dehydration, and rapid metabolic shifts further amplify arrhythmia susceptibility. The heightened adrenergic drive in HSCT resembles the autonomic imbalance seen in postoperative AF, where sympathetic surges destabilize atrial electrophysiology (29).

#### 2.1.3 Hemodynamic stress from rapid fluid shifts

Rapid intravascular volume expansion during engraftment can increase left ventricular (LV) filling pressures and atrial wall stretch. In one study, acute weight gain of ≥7% within the first week post-HSCT, especially in patients with baseline LV diastolic dysfunction, markedly increased the risk of AF. Diastolic dysfunction predisposes to left atrial and pulmonary vein stretch which may shorten the atrial effective refractory period and facilitate electrical remodeling, all of which heighten AF vulnerability (15).

#### 2.1.4 Electrolyte disturbances

Hypokalemia is a well-established risk factor for atrial and ventricular arrhythmias in the general population (30). In one study, patients who developed AF after HSCT had significantly lower serum potassium levels than controls during days 6–10 post-transplant (3.39 vs. 3.54 mEq/L, *P* = .0055), with this trend persisting from days 5–13. Although the absolute difference of ∼0.15 mEq/L is statistically significant, it is unlikely to be clinically substantial on its own (31). However, in HSCT recipients who are often exposed to multiple proarrhythmic factors even modest potassium reductions may lower the threshold for arrhythmia.

### 2.2 Post-transplant factors (>30 days)

#### 2.2.1 Immediate post-HSCT (30–100 days)

Immediate post-HSCT period factors overlap with peri-HSCT and late post HSCT. While patients are usually discharged from in-hospital setting after 30 days they would still be under close follow up in the outpatients setting till 100 days. Some of the risk factors overlap with the early period especially infections and electrolytes disturbances although the risk might be lower. During this period, patients are at risk of acute graft-vs.-host disease (GVHD) and graft rejection leading to hyperinflammatory reaction which could trigger AF (32, 33).

#### 2.2.2 Late post-HSCT (>100 days)

Chronic inflammation, chronic GVHD, therapy-induced metabolic dysregulation, and cumulative cardiotoxicity from cancer treatment converge to create both direct and indirect pathways toward arrhythmogenesis and vascular disease in HSCT survivors.

##### 2.2.2.1 Chronic inflammation and graft-versus-host disease (GVHD)

In allogeneic HSCT recipients, both acute (within 100 days) and chronic (after 100 days) GVHD create a potent proinflammatory milieu. This persistent systemic inflammation contributes to vascular injury and accelerated atherosclerosis (34). Beyond vascular disease, inflammation also promotes atrial structural remodeling and conduction heterogeneity, which together provide a substrate for AF (35). Treatment and prophylaxis of GVHD further increase this risk. Ibrutinib, a Bruton tyrosine kinase inhibitor used in hematologic malignancies and chronic GVHD, has been associated with a significantly increased risk of AF. In a meta-analysis of randomized clinical trials, ibrutinib was associated with an approximately fourfold increase in the risk of AF compared with control (36). This effect is thought to be due to off-target inhibition of kinases such as C-terminal Src kinase and disruption of PI3K-Akt signaling, which leads to atrial remodeling and abnormal calcium handling (37). Corticosteroids, calcineurin inhibitors, and other immunosuppressive drugs are associated with myocardial injury and adverse cardiometabolic effects, including hypertension, insulin resistance, and dyslipidemia, increasing arrhythmic vulnerability (38).

##### 2.2.2.2 Metabolic dysregulation

HSCT survivors are at increased risk for metabolic syndrome, driven by exposure to immunosuppressive therapy (e.g., corticosteroids, calcineurin inhibitors, mTOR inhibitors). These agents can induce insulin resistance, dyslipidemia, hypertension, and weight gain, all established risk factors for AF (39, 40). The clustering of these metabolic derangements not only raises the risk of atrial arrhythmias but also accelerates vascular disease progression, compounding long-term cardiovascular morbidity in this population.

##### 2.2.2.3 Cumulative cardiotoxicity from cancer therapy

Many HSCT recipients have prior or concurrent exposure to cardiotoxic cancer treatments. Anthracyclines, alkylating agents, conditioning regimens, thoracic radiation, and targeted therapies cause dose-dependent and often irreversible myocardial fibrosis and disruption of conduction pathways. These effects contribute directly to arrhythmogenesis and long-term cardiac dysfunction. Post-transplant, this cardiotoxic burden is compounded by GVHD therapies: for example, systemic corticosteroids not only exacerbate hyperglycemia and hypertension but also interact with existing myocardial injury to amplify arrhythmic and metabolic risk (41). Thus, cumulative cardiotoxic exposure before and after HSCT represents a key driver of late cardiovascular disease.

## 3 Risk factors for developing atrial fibrillation post transplant

**Figure 2:**
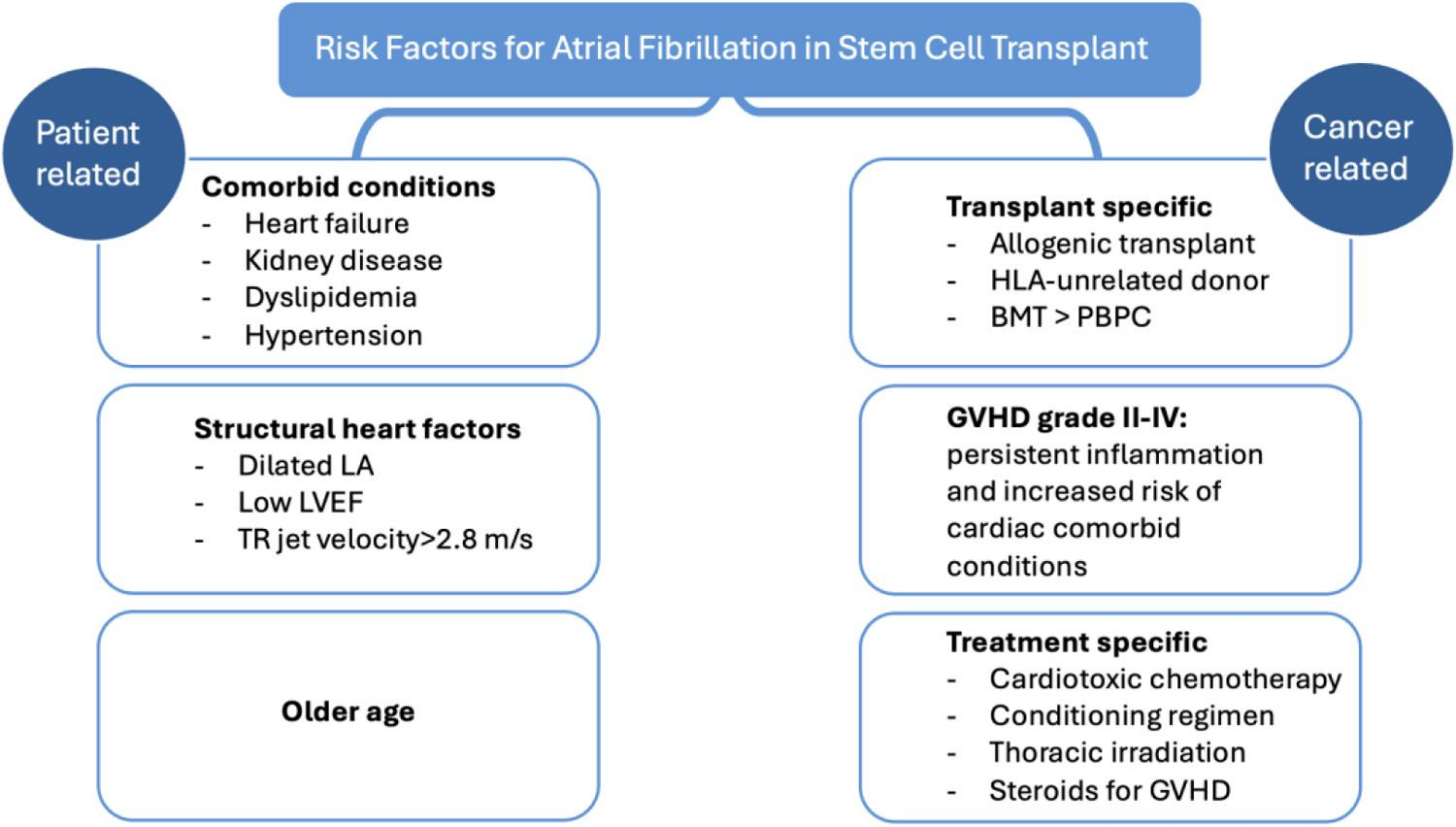
Risk factors for atrial fibrillation in patients undergoing hematologic stem cell transplant. LA, left atrium; LVEF, left ventricular ejection fraction; BMT, bone marrow transplant; PBPC, peripheral blood progenitor cells; GVHD, graft versus host disease.

Risk factors for AF following HSCT can be grouped into patient-specific and cancer/treatment-specific (Figure 2).

### 3.1 Patient–specific factors

#### 3.1.1 Age

Advanced age is a consistent and strong predictor of AF in the general population and in HSCT recipients. In a study of autologous HSCT, older age significantly increased the odds of AF [odds ratio (OR) per year: 1.14; 95% CI: 1.07–1.21] (20). Another cohort found the mean age was significantly higher in patients who developed AF (∼66 vs. 58 years; *P* = .003) (31). More recently, a retrospective single-center study (*n* = 748) confirmed that older age was an independent predictor of early post-HSCT AF (62.4 vs. 57.4 years; *P* < 0.001) (42).

#### 3.1.2 Cardiovascular comorbidities

Pre-existing cardiovascular comorbidities are important predictors of AF after HSCT. Conditions such as, chronic heart failure (HF), chronic kidney disease (CKD), pre-HSCT dyslipidemia, and hypertension have all been independently associated with an increased risk of AF in HSCT recipients (7, 14). These comorbidities contribute to structural and electrical atrial remodeling, amplify pro-inflammatory and pro-fibrotic signaling, and may interact synergistically with transplant-specific factors such as conditioning-related myocardial injury and GVHD. Moreover, patients with grade II–IV acute GVHD have been reported to have a significantly higher prevalence of hypertension, diabetes, and dyslipidemia compared with autologous HSCT survivors, further compounding long-term AF risk (43).

#### 3.1.3 Structural heart factors

Echocardiographic markers indicative of atrial remodeling significantly forecast AF risk. In allogeneic HSCT patients, lower pre-HSCT left atrial ejection fraction (<45%), diminished LA reservoir function (<39%), and elevated tricuspid regurgitant jet velocity (>2.8 m/s) were independently associated with AF risk, with odds ratios up to 12.7, 3.8, and 4.2, respectively. The presence of two or more abnormalities increased the odds by 18-fold (7). Auto-HSCT cohorts similarly showed that abnormal LV systolic function and dilated LA were associated with increased AF risk after transplant (14). GLS is a sensitive marker for early detection of chemotherapy related cardiotoxicity. A meta-analysis showed that worse absolute GLS during treatment and relative reductions from baseline were associated with a significantly higher risk of cardiotoxicity. Prognostic performance varied across studies, likely reflecting differences in GLS cutoffs, chemotherapy regimens, and underlying malignancies (44). Cardiac biomarkers, including NT-proBNP and troponins, have also been associated with adverse cardiac events in HSCT populations (45); however, their elevation may reflect subclinical myocardial injury, suggesting high sensitivity but limited specificity (46).

### 3.2 Transplant-related factors

#### 3.2.1 Type of transplant

Consistent across multiple cohorts, allogeneic HSCT is associated with a higher burden of cardiovascular complications compared with autologous HSCT (9, 43, 47). This is explained by the higher prevalence of metabolic derangements among allogeneic recipients, including hypertension, dyslipidemia, and diabetes, which are related to acute GVHD and its treatment and contribute to increased long-term cardiovascular risk (43). However, the relationship with AF appears more nuanced, as data from the same cohorts suggest similar rates of AF incidence during follow-up. In a cohort of 3,354 HSCT recipients, cumulative AF incidence was comparable between autologous and allogeneic HSCT at 100 days, 1 year, 5 years, and 10 years, with a 10-year incidence of 10.5% vs. 9.5% (*P* = 0.59) (9). Similarly, multivariable analyse from the National Inpatient Sample did not demonstrate a difference in AF incidence between transplant types (47). This suggests that AF following HSCT is largely driven by shared acute stressors—such as systemic inflammation, electrolyte disturbances, and exposure to high-dose chemotherapy and corticosteroids—rather than transplant type.

#### 3.2.2 Donor type and GVHD risk

In allogeneic HSCT, one of the key independent risk factors for AF was HLA-unrelated donor (HR: 2.20) (7). The increased risk associated with unrelated donors is likely mediated through higher GVHD incidence and its attendant inflammatory injury. Graft source can also influence arrhythmia risk. Patients receiving bone marrow transplants (BMT) have been reported to experience higher rates of arrhythmias compared with those receiving peripheral blood progenitor cell (PBPC) grafts (16). The higher arrhythmia incidence with BMT may relate to slower engraftment, prolonged hospitalization, greater fluid and transfusion requirements, and extended exposure to peri-engraftment inflammation. Similarly, recipients of T cell–depleted grafts have shown increased arrhythmia rates compared to conventional grafts, possibly due to delayed immune reconstitution and the higher prevalence of unrelated donor use in this setting (16).

#### 3.2.3 Cancer/treatment–related factors

Certain conditioning regimens appear to carry a higher arrhythmic burden; for example, high-dose melphalan in autologous HSCT and busulfan/cyclophosphamide combinations in allogeneic HSCT have been associated with increased AF incidence compared with reduced-intensity or non-alkylator-based regimens (21, 24). Prolonged steroid exposure, common in chronic GVHD management, as mentioned in the previous section, may compound this risk through sustained metabolic and hemodynamic effects (41). Prior thoracic irradiation is another important exposure with long-term cardiovascular sequelae. Mediastinal radiation can cause progressive myocardial fibrosis, valvular thickening, and conduction system injury, changes that may predispose to AF years after treatment (48). These late effects can be clinically silent until unmasked by the physiological stresses of HSCT.

### 3.3 Risk prediction

The CARE-BMT score is an externally validated pre-HSCT cardiovascular risk stratifier, but it is not AF-specific (8). It incorporates four domains (demographics, cancer-related factors, comorbidities, and laboratory parameters). However, it does not include cardiac imaging data (e.g., echocardiographic parameters), nor does it incorporate cardiac biomarkers (e.g., troponin or natriuretic peptides). Integration of cardiac biomarkers and imaging parameters in future tools would future enhance the model's prognostic accuracy. Importantly, artificial intelligence–based approaches hold promise for enhancing risk prediction and enabling more individualized assessment. There remains a need to develop a dedicated AF risk score tailored to HSCT patients to facilitate early identification, monitoring, and implement prevention strategies in high-risk patients such as prophylactic monitoring or early referral to cardio-oncology.

## 4 Management challenges

### 4.1 Management in the acute phase (within 30 days)

Patients undergoing HSCT frequently experience volume overload, which often leads to (iatrogenic) heart failure and atrial stretch, and triggering AF. Volume overload is correlated with weight gain, and each additional liter of ﬂuid administered beyond the ﬁrst 2 L was associated with 0.75 kg increase in weight over the ﬁrst 14 days. In a study by Fatema et al., a weight gain of more than 7% during the early post-transplant period was significantly associated with an increased risk of AF (15). Given this, it is essential to closely monitor fluid status assessment through daily weight measurements, jugular venous pressure (JVP), and accurate ins/outs. In patients with evidence of volume overload, prompt initiation of diuretic therapy may play a role in prevention or initial management of AF by alleviating atrial stretch and reducing neurohormonal activation.

In a study by Schulze et al., patients who developed AF during stem cell transplantation (SCT) were found to have lower serum potassium levels compared to matched controls (31). Hypokalemia is a well-recognized pro-arrhythmic factor that can promote ectopic atrial activity and trigger arrhythmias, particularly in the context of myocardial stress or inflammation (49). Therefore, prompt recognition and correction of electrolyte disturbances-especially potassium and magnesium-is essential in the acute management of AF following HSCT.

#### 4.1.1 Anticoagulation

Decision to anticoagulate or not is a complex in patients with cancer as these patients are at higher risk of thrombosis, and bleeding. However, in the acute setting, they have profound bone marrow suppression with significant thrombocytopenia, and thus, often, it is not feasible to initiate anticoagulation even if the risk of thrombosis is elevated. While these patients are at elevated risk of thrombosis due to multiple factors, including malignancy itself and chemotherapy. Concomitant thrombocytopenia may mitigate this risk, as suggested by a meta-analysis in patients with AF showing that those with thrombocytopenia had a significantly lower risk of ischemic stroke or systemic embolism compared with those without thrombocytopenia (OR: 0.79, 95% CI: 0.69–0.91) (50).

#### 4.1.2 Rate vs. rhythm control

In a study by Singla et al. involving 92 patients who developed AF or atrial flutter (AFL) following bone marrow transplantation, only 7 patients (7.6%) required electrical cardioversion, while none received pharmacologic cardioversion. The majority (82/92) were managed with rate control agents (beta-blockers or calcium channel blockers) and 88% (81/92) had converted to sinus rhythm by hospital discharge. Notably, this study did not report outcomes by treatment strategy or rates of thromboembolism (21). Overall, direct comparisons between rate and rhythm control in this population are lacking, and management strategies remain variable across institutions.

According to a scientific statement by American Heart Association on cardiovascular disease management in patients undergoing HSCT, in the acute setting, they favor rate control over rhythm control, as these patients have profound thrombocytopenia and cannot safely receive anticoagulation (10). Verapamil and diltiazem, both CYP3A inhibitors, have drug-drug interactions with chemotherapy, and negative inotropic effects. As a results beta blockers might be favored as a rate control medication in the context of HSCT (51).

However, this paradigm warrants reconsideration in light of emerging data on the time-dependent nature of thromboembolic risk. Although spontaneous and active cardioversion (pharmacologic or electrical) appear to carry comparable embolic risks (52, 53), thromboembolism is closely related to AF duration. Data from the FinCV study demonstrate that earlier cardioversion is associated with lower thromboembolic risk, with rates of 0.3% when performed within 12 h compared with 1.1% beyond 12 h (54). These findings suggest that minimizing time in AF may be a key modifiable factor, particularly in patients who cannot receive anticoagulation. In this context, a purely “wait-and-see” strategy may be suboptimal in HSCT patients. Because anticoagulation is often not feasible, allowing spontaneous conversion may prolong AF duration and increase thrombotic risk without clearly reducing embolic events compared with active cardioversion. Accordingly, early rhythm control, ideally within 12 h of AF onset, may be a reasonable approach when onset is well defined, as is often the case in hospitalized transplant patients. Amiodarone is often the preferred option in this setting as most alternative antiarrhythmics carry higher risk of QT prolongation, and several are contraindicated in patients with underlying structural heart disease.

**Figure 3:**
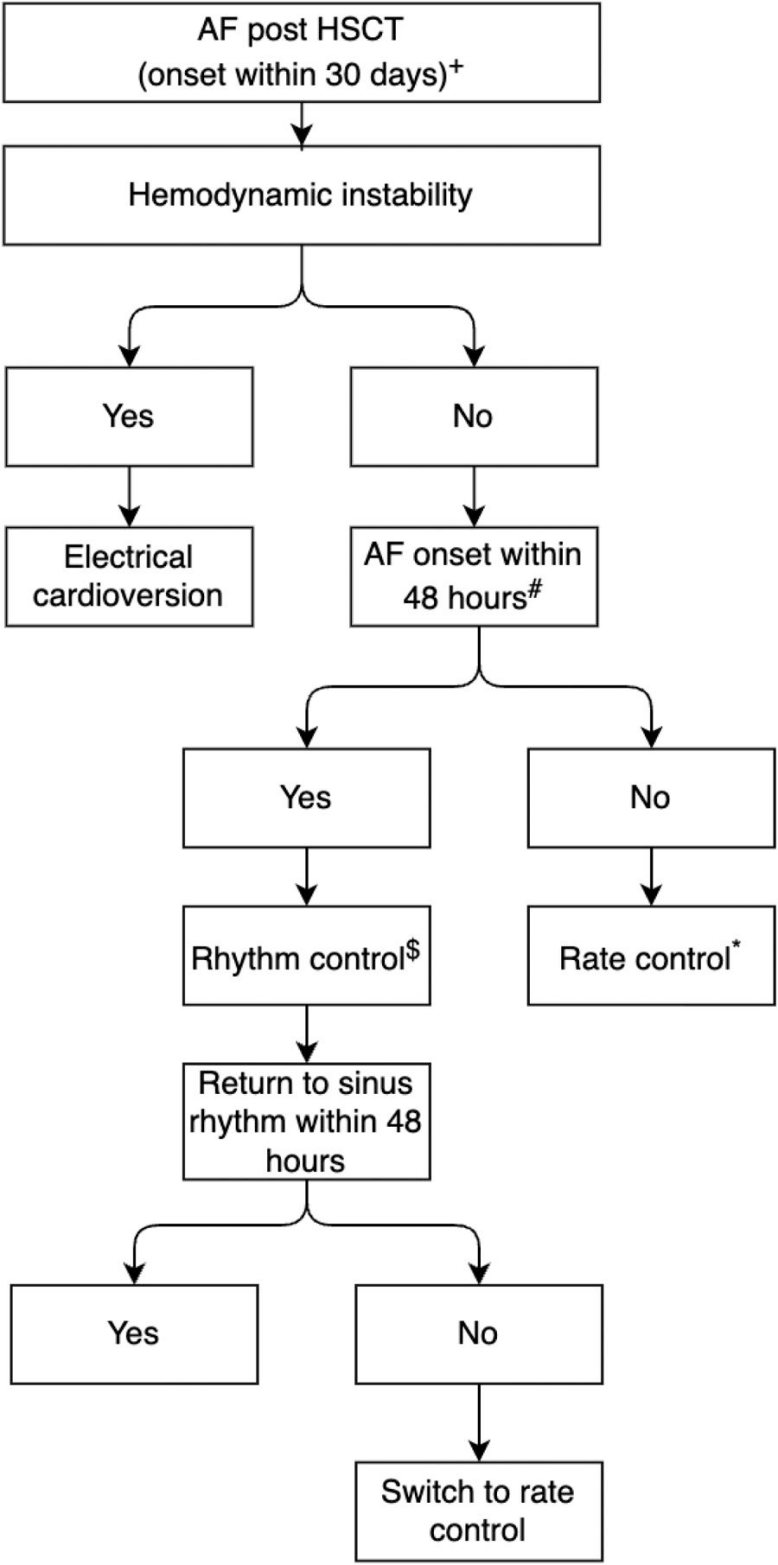
Proposed management algorithm for New-onset atrial fibrillation after HSCT (≤30 days). **Footnote**: **^+^**Profound thrombocytopenia—no anticoagulation. ^#^Ideally within 12 h. ^$^Electrical or pharmacological cardioversion (e.g., amiodarone). *Preferably beta-blockers. AF, atrial fibrillation; HSCT, hematopoietic stem cell transplant.

Importantly, this strategy should be time limited. Beyond 48 h, cardioversion without anticoagulation becomes guideline-discordant in the absence of transesophageal echocardiographic exclusion of atrial thrombus. Moreover, continued use of antiarrhythmic therapy may result in delayed, unprotected cardioversion. Accordingly, if sinus rhythm is not achieved within 48 h, patients should be transitioned to rate control (Figure 3).

### 4.2 Long term management of AF

The onset of AF following HSCT is often attributed to transient factors, such as the peri-transplant period, inflammatory stress, volume shifts, or the use of medications known to promote AF. Traditionally, such episodes have been considered isolated events with limited long-term significance. However, emerging evidence suggests that this assumption may not hold true, as AF may reflect the presence of an underlying atrial substrate predisposing to arrhythmia recurrence. In fact, AF recurrence rates after an initial post-HSCT episode are high, with 25% of patients experiencing recurrence within the first month (21), and 46% within one year of transplantation (31). These findings highlight the need for careful long-term monitoring and reassessment of the notion that post-HSCT AF is purely transient or benign.

#### 4.2.1 Anticoagulation

##### 4.2.1.1 Thromboembolic risk

The decision to initiate OAC in patients who develop AF after HSCT is particularly challenging due to the concurrent high risk of both thrombosis and bleeding in this population. Although malignancy and cancer therapies (e.g., lenalidomide) (55) are well-recognized risk factors for thrombosis, validated risk prediction models for stroke in AF-including CHA₂DS₂-VASc (56), ATRIA (57), and GARFIELD (58) excluded patients with malignancy from their cohorts. As such, these tools may underestimate stroke risk in cancer patients with AF and they may not be applicable in this population. In Chang et al., approximately 10% of patients with AF developed stroke during follow-up, corresponding to an incidence rate of 143 per 1,000 person-years, exceeding even the highest risk strata defined by the CHA₂DS₂-VASc score. Anticoagulation was rare used in this cohort, which may partially explain the high observed stroke rate (7). In contrast, Bolaji et al. reported a lower stroke incidence of approximately 3% among patients with AF (13), while Zaghlol et al. reported a 1.2% incidence of arterial thromboembolic events at 3 months (5). Differences in follow-up duration and the lack of consistent reporting on anticoagulation use limit direct comparison across studies.

Overall, the true incidence of stroke following AF in HSCT populations remains uncertain. The absence of consistent data on anticoagulation further limits interpretation. Prospective studies are needed to better define thromboembolic risk and clarify the role of anticoagulation in this population.

##### 4.2.1.2 Bleeding risk

Patients with hematologic malignancies are at increased risk of bleeding, particularly due to bone marrow suppression associated with the malignancy itself or with ongoing maintenance cancer therapy. Traditional bleeding risk scores, such as HAS-BLED (59) and ATRIA (57), do not account for active malignancy, limiting their applicability in this population. Although the HEMORR₂HAGES score does include malignancy as a risk factor, it is less commonly used in clinical practice (60). Importantly, all three tools demonstrate limited discriminatory ability to differentiate between patients who will or will not experience bleeding events (61). Moreover, several components of these scores-such as age and prior stroke-also correlate with an increased risk of thromboembolism, complicating their interpretation in anticoagulation decisions. In this context, direct clinical assessment of bleeding risk, including the presence of absolute contraindications to anticoagulation (e.g., platelet count <50,000/µL, active or recent major bleeding), is often more useful than relying on these scoring systems (62).

Despite the elevated stroke risk observed in the study by Chang et al., anticoagulation was rarely utilized (7). In another cohort, the mean CHA₂DS₂-VASc score was 2, yet only 6 out of 15 patients received anticoagulation (31), reflecting a potential under-treatment of thromboembolic risk due to limitations of current risk stratification tools in cancer patients and because of the higher risk of bleeding in this population.

According to 2023 ACC/AHA/ACCP/HRS Guideline for the Diagnosis and Management of Atrial Fibrillation, decision for OAC is not based on duration of AF but rather annual risk of stroke. Patients with an annual stroke risk >2% should receive anticoagulation (Class I recommendation), while it is reasonable in those with a 1%–2% risk (Class IIa) (51).

Importantly, population-based studies suggest that the benefits of anticoagulation generally outweigh the risks of bleeding, even in patients considered high bleeding risk (63, 64). Therefore, bleeding risk scores should not be used in isolation to withhold therapy, but rather to identify modifiable risk factors and guide clinical monitoring.

When the decision is made to initiate anticoagulation, direct oral anticoagulants (DOACs) may be favored over warfarin. Posthoc analysis of randomized trials comparing DOACs with warfarin in patients with AF and cancer have demonstrated at least noninferiority of DOACs (65–67); These analyses primarily included patients with solid tumors and only a small proportion with hematologic malignancies, which may limit the direct applicability of these findings to the HSCT population. Nevertheless, given their more predictable pharmacokinetics, fewer drug–drug interactions, and reduced need for monitoring compared with warfarin (68), DOACs represent a practical anticoagulation strategy in patients with AF post HSCT.

#### 4.2.2 Rate vs. rhythm control

Although maintaining sinus rhythm may be desirable, particularly given the prothrombotic state associated with malignancy and HSCT, as well as the typically shorter duration of AF, both rate and rhythm control are acceptable strategies. The approach should be individualized based on patient age, symptom burden, anticoagulation status, and comorbidities. Rhythm control medications carry risks of QT prolongation and drug–drug interactions in the setting of chemotherapy, antifungals, and supportive medications (e.g., ondansetron). Among available options, amiodarone is generally preferred over dofetilide or sotalol, given its lower propensity to cause QT prolongation and torsades de pointes, particularly in the context of electrolyte disturbances (hypokalemia and hypomagnesemia), which are common in this population (51). For rate control, beta-blockers are generally preferred due to their favorable cardiac outcomes, especially in the setting of Cancer Therapy-Related Cardiac Dysfunction (CTRCD). In contrast, verapamil and diltiazem have limited long-term cardiovascular benefit and may pose a risk of drug–drug interactions through CYP3A4 inhibition, affecting the metabolism of multiple chemotherapeutic agents and DOACs (6).

Beyond the acute phase, if AF persists, catheter ablation may be considered in selected patients, particularly those with ongoing symptom burden or intolerance to medical therapy, considering patient comorbidities and cancer-related factors (e.g., disease status and prognosis) (51). Successful catheter ablation may reduce thromboembolic risk and allow for simplified antithrombotic strategies. Emerging data from recent trials, including ALONE-AF and OCEAN, suggest that no antithrombotic or aspirin alone is noninferior to oral anticoagulation for thromboembolic prevention following successful ablation, with a lower risk of bleeding (69, 70). This may be particularly relevant in patients with hematologic malignancies, in whom bleeding risk is high and anticoagulation is often not feasible. In addition, catheter ablation may reduce reliance on long-term antiarrhythmic therapy, thereby minimizing potential drug–drug interactions with chemotherapeutic agents.

However, no randomized trials have evaluated catheter ablation in patients with hematological malignancy. Available observational data are limited and report inconsistent findings, with some studies suggesting higher rates of complications, including infection and bleeding (71–73) Importantly, these studies are subject to significant selection bias and confounding and they don't report on the procedural success rates, limiting the ability to draw definitive conclusions.

## 5 Limitations of current evidence and future directions

Most available evidence is derived from retrospective cohorts, resulting in overall low certainty due to inherent limitations, including confounding and selection bias. Additionally, most studies are single center, with significant variability in HSCT practices, treatment protocols, and follow-up duration, further limiting generalizability and contributing to significant variability in reported outcomes. There is a substantial evidence gap regarding optimal management strategies. Data guiding rate vs. rhythm control and antithrombotic therapy in HSCT patients are extremely limited, and extrapolation from the general AF population may be inappropriate given the unique balance of bleeding and thrombotic risks in this population.

Although, the body of literature on AF following HSCT is growing, there remains a critical need for prospective, multicenter studies and registries to better define its predictors, clinical impact, and management. In particular, improved risk stratification is needed, including the development of predictive models, potentially leveraging artificial intelligence approaches, to identify patients at highest risk for AF.

## 6 Conclusion

AF is an increasingly recognized as a complication after HSCT, arising from a confluence of mechanisms related to cancer and treatment. Traditional cardiovascular risk factors further amplify susceptibility in this population. Despite improved survival in HSCT, strategies to predict, prevent, and manage AF remain limited. Current evidence is derived largely from retrospective analyses, underscoring the need for prospective studies to assess incidence of thromboembolic events and comparing outcomes of different treatment approaches (rate and rhythm control).
